# Trends in Degradable Mesoporous Organosilica-Based Nanomaterials for Controlling Drug Delivery: A Mini Review

**DOI:** 10.3390/ma13173668

**Published:** 2020-08-19

**Authors:** Vanessa Poscher, Yolanda Salinas

**Affiliations:** Institute of Polymer Chemistry (ICP) and Linz Institute of Technology (LIT), Johannes Kepler University Linz, Altenberger Straße 69, A-4040 Linz, Austria; vanessa.poscher@jku.at

**Keywords:** mesoporous organosilica nanomaterials, degradable, periodic mesoporous organosilica nanomaterials, stimuli-responsive molecular gates, drug delivery, nanomedicine

## Abstract

The last few years of enhancing the design of hybrid mesoporous organosilica nanoparticles has allowed their degradation under specific pathologic conditions, which finally is showing a light in their potential use as drug delivery systems towards clinical trials. Nevertheless, the issue of controlling the degradation on-demand at cellular level still remains a major challenge, even if it has lately been addressed through the incorporation of degradable organo-bridged alkoxysilanes into the silica framework. On this basis, this mini review covers some of the most recent examples of different degradable organosilica nanomaterials with potential application in nanomedicine, from degradable non-porous to mesoporous organosilica nanoparticles (MONs), functionalized with responsive molecular gates, and also the very promising degradable periodic mesoporous organosilica materials (PMOs) only consisting of organosilica bridges.

## 1. Introduction

The last few years of developing and designing the ideal drug delivery system have faced crucial challenges. The initial goal is to use the specific drug in a certain concentration to be delivered over a specific period of time, according to the application. If the concentration of drug delivered is too high, the treatment can cause an overdose, while if the concentration released is too low, a risk of an incomplete treatment exists, inducing, in the worst scenario, the death of the patient. Therefore, it is not an easy task to achieve an effective system concentration without reaching toxicity levels, due to the large drug amounts required during illness treatments, which in some cases is also affected by the bioavailability of the drugs. Secondly, the trend in the new drug delivery system’s preparation is to have a regulated cargo delivery based on a support that may degrade afterwards and can be excreted from the human body. In order to achieve that challenge, the drug delivery system should break into smaller molecules avoiding agglomeration that may lead to undesired side-effects [1].

Silica materials for drug delivery applications can be prepared with and without pores. Both materials are synthesized utilizing the well-known Stöber process. In the case of porous silica particles, a surfactant, which acts as a structure directing agent, has to be used. The surfactant forms micelles, and the silica structure grows around those micelles, resulting in porous particles. These pores can be utilized to fill them with different molecules, e.g., drugs, which can be released after a specific stimulus is applied. Another advantage is the large surface area of porous materials, which can be advantageous for either the functionalization of the materials using different molecules or they can also help with the degradation of the materials. The non-porous silica particles are also synthesized using the sol-gel method, but in this case no surfactant is utilized. Nevertheless, their ability for drug delivery is limited compared to the porous nanoparticles.

**Figure 1:**
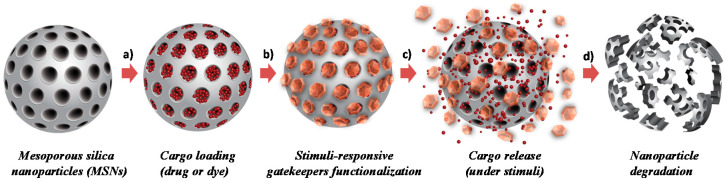
Schematic representation of mesoporous silica nanoparticles (MSNs) (**a**) loaded with selected dye or drug molecules, followed by (**b**) functionalization of the silica surface with gatekeepers controlling the (**c**) release of the cargo on-demand under specific stimuli and desired final (**d**) degradation upon completion of their cargo release.

Among other types of drug carrier materials, mesoporous hybrid organosilica nanomaterials (i.e., M41S mesoporous family or Santa Barbara Amorphous, SBA type materials) have set a precedent during the last few decades, presenting a specific combination of silicates and organic units, while providing unique porosity that enhances the properties of conventional pure Si-O-based materials [2]. They can be designed with uniform morphology and size, tunable composition, and a stand-out chemical stability of the Si-C bonds, added up to their unique non-connected parallel pores. Moreover, a facile chemical modification of their silane-based surface with a wide range of responsive organic or inorganic moieties is possible, for example to act as pore-blockers controlling the drug released. Known by the research community as molecular gates, these responsive units allow the control of the closing and opening of the pore outlets on-demand upon applying specific stimuli [3]. By using this smart functionalization approach, the entrapped drug is not allowed to release unless a specific external stimulus induces the removal of the cap or the change in conformation of the responsive functionalized units opening the gates, thereby setting free the cargo (see Figure 1). In addition, because of the well-demonstrated robustness and inertness of the silica, these materials are well-demonstrated as biocompatible in dosages required for in vitro and in vivo studies [4]. These features of the mesoporous silica nanoparticles (MSNs) caused them to be stand-out candidates in the design of nanomedicine formulations.

The structure of MSNs has been demonstrated to be susceptible to the hydrolytic breakdown of the siloxane (Si-O-Si) in aqueous solutions into biocompatible and excretable orthosilicic acid (Si(OH)_4_) [5]. Nevertheless, their complete degradation in body fluids after the drugs are delivered under controlled conditions is still an essential and challenging requirement for therapeutic applications [6,7,8]. Therefore, to address this issue, the design of silica-based nanoparticles incorporating organo-bridged alkoxysilanes within the mesoporous silica frameworks that can degrade under specific controlled conditions may be an essential feature for their future clinical translation [9].

**Figure 2:**
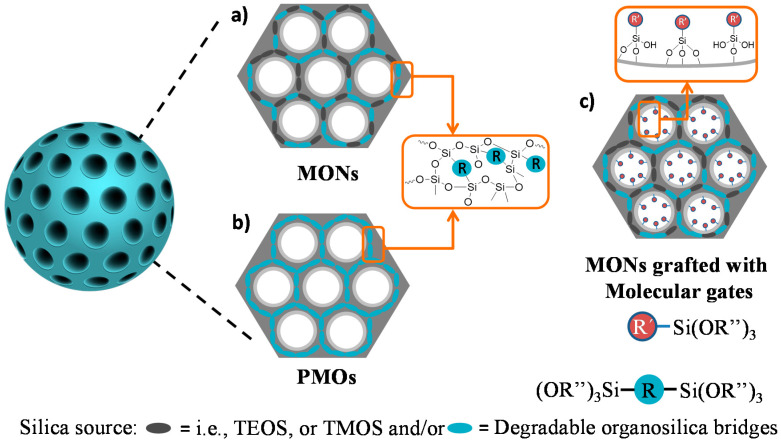
Schematic representation of degradable (**a**) mesoporous organo-bridged silica nanoparticles (MONs); (**b**) periodic mesoporous organosilica (PMOs) nanoparticles, prepared with degradable organosilica alkoxysilane bridge units as unique silica source, both PMOs and MONs obtained after surfactant extraction; and (**c**) possible grafting of stimuli-responsive molecular gates onto the surface of MONs for controlling drug delivery.

In the case of MSNs, only a silicon alkoxide precursor (e.g., classical silane precursor (i.e., tetraethyl orthosilicate, TEOS, or tetramethyl orthosilicate, TMOS)) is used for the synthesis [10], but if mixed with an organosilane unit during the preparation, mesoporous organosilica nanoparticles are formed. In principle, there are two kinds of organosilica materials (see Figure 2), differing by the type and ratio of functional organic groups incorporated into the silica walls. On the one hand, the use of 100 % of one or more bis- or multi-bridged organosilane precursors, when no additional silane source is present, leads to the formation of periodic mesoporous organosilicas (PMOs) (see Figure 2b). This family of mesoporous silica materials was discovered in 1999, in which porous frameworks are obtained from organo-bridged alkoxysilanes in the presence of structure-directing agents by the sol-gel process [11,12]. On the other hand, the use of less bridged organic groups together with the classical TEOS or other silane precursors leads to mesoporous organo-bridged silica nanoparticles (MONs) (see Figure 2a) [13,14]. Even today, there is still not a homogeneous consensus regarding the specific nomenclature for differently synthesized organosilica materials. The nomenclature we used in this mini review to categorize porous organosilica nanoparticles is the general standard used by most other groups [15].

In general, the synthesis of MONs and PMOs is very similar to the traditional mobile crystalline material MCM-41 materials (M41S-type material), following the condensation of hydrolysable mono- or bis-silane units around an arrangement of micelles of a pore-forming agent (i.e., surfactant cetyltrimethylammonium bromide, CTAB). In the bis-silane units (R”O)_3_Si-R-Si(OR”)_3_, R” are usually methoxy or ethoxy groups, while R stands for organic functional linkers between the silicon atoms (see Figure 2a). In this respect, the organosilica units condense around the pre-formed surfactant micelles and the walls of the silica network are formed [16]. After an ageing period, the surfactant can be removed by extraction, leaving the highly porous material. The homogeneous and diverse chemical pore nature makes them unique systems that are currently being applied more often in nano-biomedicine [17]. In fact, these prolific materials are known to accomplish many biomedical requirements, such as improved hydrothermal and hydrolytic stability, good colloidal dispersity, adjustable biodegradability, and high loading capacity towards specific drugs, framework-induced stimuli-responsive release behavior, good biocompatibility, and particle sizes in the nanoscale range, all providing more efficient theranostic solutions if chosen the appropriate bridging groups. This class of materials can introduce organic functional groups that can be cleaved under specific physiological conditions (for example, if enzymatic degradation features are added via cleavable amide-bridges) or other types of degradable linkers, that lead to the complete disintegration of the silica-based particles (as shown above in Figure 1d) [18,19]. Nevertheless, the selection of very complex organic groups may disturb the periodic and uniform mesostructured order, which is a crucial factor to keep in mind. Thus, the importance of the right choice of organic cleavable bridges, which can promote degradation, and their homogeneous distribution in the silica structure, was reported [20].

At the present time, maintaining the possibility of structural control while increasing the proportion of soft organic components for effective and controlled degradation is still in its infancy. Furthermore, true biodegradation requires complete degradation within the time frame of the release application, which is relatively short for a pharmaceutical product, and consequently, the use of short and simple organic bridges can be limited [21]. Therefore, a new paradigm is required to obtain MONs and PMOs that are structurally stable during the synthesis but are also truly degradable in a suitable timeframe after application. Moreover, such materials can be prepared as gated-like nanosystems by grafting responsive molecules that control the on-demand release of a payload (see Figure 1b–d) to finally have degradable variants, which allows the disintegration of the whole nanosystem. Although such degradable materials could be used in many applications, a very appealing field in which there is an acute requirement for degradable materials is nanomedicine, as mentioned above. In the design of an ideal drug delivery system, an important effect to avoid is the premature release of potentially toxic payloads, which not only leads to lower efficacy but also to possible severe side effects. In the next few years, the grafting of responsive molecular gates onto the surface of the nanoparticles designed for degradability (see Figure 2c) is envisioned to be much further developed.

In this mini review, we have collected publications from the last 5 years to the beginning of 2020, which we considered to be very potent examples reported on organosilica-based porous and non-porous nanomaterials used for controlled and sustained release under external stimuli, leading to their final degradation at the cellular level. This mini review will also highlight the possible post-modification of the surface of degradable MONs with molecular gates, introducing the advantageously controlled cargo transport out of the pores upon applied stimuli, of extreme interest in future theranostic applications [22].

## 2. Degradable Non-Porous Organosilica Nanoparticles for Drug Delivery

Monodisperse, compact, non-porous silica-based nanoparticles, which are easy to synthesize present a robust structure for drug delivery purposes, and furthermore, a controllable size and composition [23]. They are commonly prepared using the sol-gel method, which was developed in 1968 by Stöber and co-workers [24]. This method followed the well-known bottom-up approach to fabricate silica particles under a wide control of sizes (ranges from 50 nm to 1–2 μm) and a spherical morphology according to the reaction parameters selected [25]. However, this method had some limitations, which are addressed by modifying the original synthesis in order to achieve both monodispersity and degradability, but avoiding the use of surfactants during their preparation [26]. Altogether, recent developments on tailored sol-gel silica nanoparticle’s preparation, characterization, and surface modification, has shown its use in different appealing areas of technology, among them, in nanomedicine.

### 2.1. Non-Porous Nanoparticles Containing Sulphide or Oxamide-Based Organosilane Bridges

**Figure 3:**
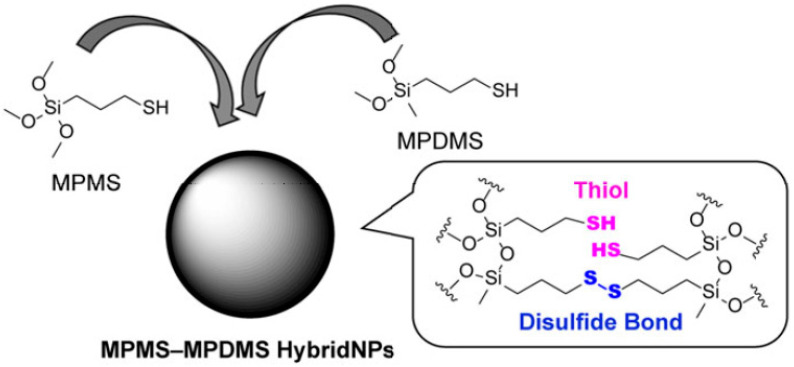
Structure of thiol-organosilica hybrid nanoparticles (MPMS-MPDMS HybridNPs) containing two types of disulfide bridges as silane sources. Reproduced from [29] (https://doi.org/10.1557/jmr.2018.501), with permission of The Royal Society of Chemistry.

A very trendy approach for the preparation of degradable organosilica nanoparticles is the introduction of biodegradable disulfide (S-S) groups via a co-condensation approach, with the purpose of intracellular degradation upon exposure to reducing agents such as glutathione (GSH). It is notorious that there is a difference in the GSH concentration between tumor tissues (2–10 mM) and normal tissues (below 0.002 mM) [27]. In this regard, Mekaru H. and co-workers used uniquely thiol-containing precursors in the preparation of degradable silica-based nanoparticles [28]. Here, (3-mercaptopropyl) trimethoxysilane (MPMS) or (3-mercaptopropyl) methyldimethoxysilane (MPDMS) were selected to form three or two siloxane bonds inside the silica support, respectively. These thiol-organosilica nanoparticles (NPs) degraded through the reduction of the disulfide bonds to thiol groups in the presence of the reducing agent glutathione (GSH). Interestingly, only in the case of particles prepared using two siloxane bond containing molecules, degradation under the influence of GSH followed, where the high GSH concentration mimicked intracellular reducing conditions. In another example, Doura T. and co-workers prepared non-porous disulfide bridges containing organosilica nanoparticles, also using MPMS and MPDMS as silane sources (see Figure 3) [29]. Different hybrid nanoparticles with increasing contents of MPDMS showed the effect of increasing amounts of disulfide bonds on the degradation rate. Consequently, higher amounts of GSH were required to induce the degradation of these stimuli-responsive disulfide bond-containing silica nanoparticles. Furthermore, these MPMS-MPDMS (25:75) based NPs were doped with rhodamine B and the GSH-responsive release of dye from the NPs demonstrated their potential as responsive drug carriers. Moghaddam S. P. H. et al. also presented a system responsive to redox changes by synthesizing sulfide groups-containing organosilica nanoparticles, which degrade at higher GSH concentrations present in tumor tissue [30]. Particles with different diameters and compositions were prepared by incorporating disulfide- or tetrasulfide bonds in the silica framework. In this case, mixtures of TEOS and bis[3 -(triethoxysilyl)-propyl]disulfide or bis[3-(triethoxysilyl)-propyl]tetrasulfide, or the disulfide-containing molecules alone, were used as silane sources. It is worth noting that using the mixtures for the sol-gel process, mesoporous organosilica nanoparticles (MONs) could be also obtained, while if the sulfide-containing silane sources were used without any TEOS, the process resulted in non-porous organosilica nanoparticles. Although these non-porous nanoparticles showed very appealing features, their application as drug delivery systems has shown their limitations in comparison with the mesoporous drug carriers [31].

**Figure 4:**
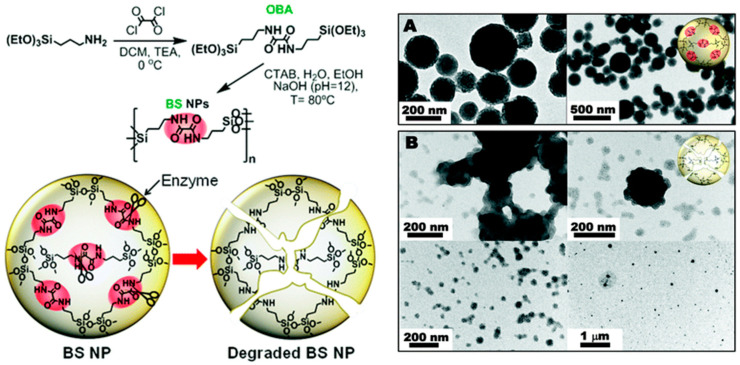
Particle preparation using oxamide cleavable groups and enzyme-triggered cleavage of amide bonds causing degradation of the silica-based nanoparticles. TEM images of the bridged silsesquioxane (BS) composite nanomaterials in phosphate buffer solution, PBS (**A**) and in trypsin-PBS buffer (**B**) after 24 h of enzymatic degradation. Adapted from [18] (https://doi.org/10.1039/C5NR03065J), with permission from The Royal Society of Chemistry.

The degradability features of non-porous silica-based nanoparticles may have a great potential for the delivery of drugs and/or as imaging tools, importantly, if their degradation occurs shortly after the accomplishment of their purpose inside the biological environment. Of course, the nanomaterial itself and its small side products after degradation are also expected to have very low cytotoxicity, being released into the urine to leave the human body [32]. In 2015, the group of Khashab published non-porous biodegradable bridged silsesquioxane nanoparticles with an unusually high organic content of oxamide-bridged alkoxysilanes as biocleavable groups (ca. 50%) incorporated into the siloxane framework via sol-gel reaction. This research group was able to achieve monodisperse nanoparticles with diameters <200 nm, which degraded in the presence of the enzyme trypsin through the cleavage of amide bonds (see Figure 4) [18]. Moreover, fluorescein isothiocyanate moieties were added to allow the use of these nanocomposites for in vitro imaging applications.

### 2.2. Non-Porous Silica Nanoparticles Containing Other Degradable Organo-Bridged Alkoxysilanes

The introduction of other potential degradable organic units into the silica matrix, such as degradable polymer units, may drive the formation of novel degradable organosilica nanoparticles. Recently, our group reported the preparation of surfactant-free organosilica compact nanoparticles using this sol-gel process, but avoiding the use of a “classical” silane source, such as TEOS [23]. In particular, novel hybrid phosphazene-based silica nanoparticles were synthesized with two different silane group containing cyclomatrix phosphazenes and linear polyphosphazene units. For that, the silane group containing (3-mercaptopropyl)trimethoxysilane was linked via a thiol-ene photoreaction to the double bonds forming the cyclic and the linear polymeric-based organo-bridged alkoxysilanes. This type of polymer was selected due to its potential well-known degradation into harmless bioproducts [33]. The framework should disintegrate into small, however not yet proven, easily excretable molecules for medicine applications [34].

**Figure 5:**
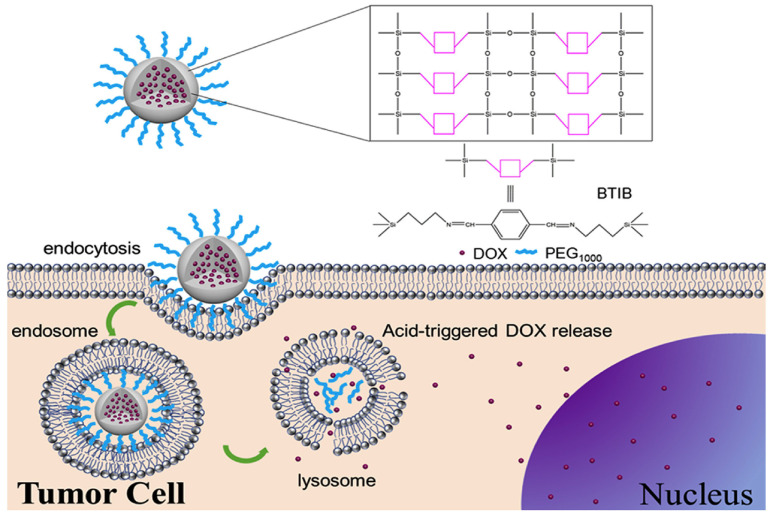
Illustration of the nanoparticle’s structure and their entering in the tumor cells by endocytosis. This process is followed by the acid-triggered drug release and nanoparticles biodegradation. Reproduced from [37] (https://doi.org/10.1016/j.jddst.2019.101450) with permission from Elsevier.

The drug carrying possibilities of these compact silica-based nanomaterials rely on: (i) the functionalization of the drug molecules to the −OH groups from the silica surface with an extra inconvenient modification according to the chemical structure of the drugs [23,35]; (ii) drugs to be physisorbed on the silica nanoparticle’s surface, which will also depend on the available active surface area correlated to the nanoparticle’s diameter; or (iii) the incorporation of the payload or drug doping within the matrix during the nanoparticle’s preparation [36]. Therefore, the challenge of preparing degradable nanoparticles that can control the degradation profile in order to obtain an effective drug delivery all-in-all in an aqueous bioenvironment and in the absence of additional stimuli or reagents is still in development. Very recently, S.-Y. Peng and co-workers constructed non-porous biodegradable organosilica nanoparticles used for drug delivery, and later on degraded by endosomal trigger (see Figure 5) [37]. Here, the monomer bis(3-ethoxysilane)propylimylamine methyl benzene (BTIB) was prepared through the condensation of p-benzaldehyde and (3-aminopropyl)triethoxysilane, which was afterwards used for organosilica nanoparticle preparation, followed by corresponding hydrolysis and crosslink reactions.

These particles consisted of benzene groups and acid-labile imine bonds, which were able to carry drug molecules by the hydrophobic interaction between the benzene functionality and the drug units (in this case, classical chemotherapeutic drug doxorubicin). The authors also coated the nanoparticles with the hydrophilic poly(ethylene glycol) (PEG), which is a common strategy selected for prolonging the NPs circulation time [38]. The imine bond was highly sensitive to the slightly acidic environment found in the tumor cell’s endosome (pH~5.0), leading to the release of the drug followed by the breaking of the particle’s framework in low molecular weight fragments, easily excreted from the human body. As a result, this reported example demonstrated the possibility to incorporate the drugs inside the nanoparticles, and be released after degradation.

For the delivery of anticancer drugs, the degradation of silica nanocarriers in an aqueous environment might be attractive. In that direction, non-porous water-degradable silica nanoparticles were prepared using a bridged sorbitol-based silsesquioxane precursor containing carbamate linkages [39]. Here, the organosilane-bridge containing precursor was prepared from sorbitol moieties reacted with (3-isocyanatepropyl)triethoxysilane (ICPTES). The resulting molecules with two carbamate functionalities were reacted by co-condensation reactions with TEOS to obtain these ICPTES-sorbitol silica nanoparticles. Remarkably, these non-porous silica nanoparticles became porous and started to degrade upon prolonged exposure to an aqueous environment through the hydrolysis of the carbamate bonds. These NPs showed different hydrolysis rates depending on the acidic character of the environment, which is of interest in oral-based drug delivery and application.

## 3. Degradable Mesoporous Organo-Bridged Silica Nanoparticles (MONs) for Drug Delivery

Over the years, the introduction of pores into silica-based nanoparticles has demonstrated their enhancement of potential applications, such as drug delivery systems (DDS). As with the traditional TEOS-based mesoporous silica nanomaterials, porous drug delivery systems based on organosilica nanoparticles also present interesting advantages, such as (i) biocompatibility, (ii) high loading capacity, (iii) higher protection of the cargo or drug molecules, and (iv) possible control of the cargo release via surface modification. Furthermore, the high organic content in MONs enables tailored framework degradation, very much demanded in biomedical future uses. In order to obtain pores, a structure directing agent (such as CTAB) is required. After surfactant removal, the pores are available for cargo uploading. The most remarkable addition to classical mesoporous-based nanomaterials is that mesoporous organosilica nanoparticles may overcome the current problems associated with the lack of biodegradability, which has been the major hurdle in their translation into clinics. In this chapter, we have selected the most recent examples related to degradable MONs applied in drug delivery, also covering a few recently reported gated-MONs for the controlled delivery of drugs in cancer cells.

### 3.1. MONs Containing Biocleavable Oxamide or Sulphide-Based Organosilane Bridges

Notably, Croissant and co-workers prepared mesoporous organosilica nanoparticles, using only oxamide-phenylene bridges containing precursors for the sol-gel reaction. To obtain these nanomaterials, which were biodegradable in the presence of proteins, no additional silane source was utilized [31]. These particles were prepared through the co-condensation of 1,4-bis(triethoxysilyl)-benzene and (*N,N*′-bis(3-(triethoxysilyl)-propyl)oxamide). Through this method, the oxamide functional units were homogeneously distributed within the organosilica scaffold, leading to 100 nm nanoparticles with high surface areas of 850 m^2^ g^−1^ (close to traditional MSNs). Moreover, the exceptionally high organic content obtained (about 50 wt. %) allowed the loading of the pores with both hydrophilic doxorubicin (DOX) hydrochloride and hydrophobic camptothecin. These MONs presented much higher drug payloads than the non-porous ones (described in Chapter 2), and certain gatekeepers are thought to be required for controlling the release upon a specific stimulus. Compared to other mesoporous silica systems reported in this example, the drugs remained in the pores of the oxamide-phenylene-containing silica network from pH 7.4 to 5 without the need of pore capping and showing a unique zero premature leakage. After specific amino acids were cleaved by trypsin into carboxylate and ammonium derivatives, the particle’s degradation caused the drug release.

**Figure 6:**
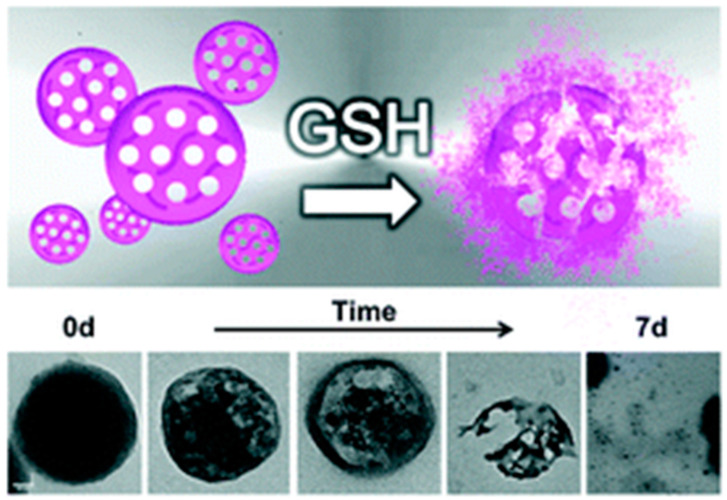
Illustration of self-destructive redox-responsive hybrid mesoporous silica nanoparticles and TEM images of ss-NPs during the undergoing of glutathione (GSH) (10 mM) reduction for 7 days. Image reproduced from (https://doi.org/10.1039/C5NR09112H) [40] with permission from the Royal Society of Chemistry.

In the construction of redox-responsive organic-inorganic hybrid mesoporous organosilicas, disulfide-containing precursors were selected as bis-silylated organosilica precursors. Very promising, redox-responsive hybrid mesoporous organosilica nanoparticles containing disulfide (S-S) bridges were published in 2016 by the research group of Luisa De Cola [40]. These degradable nanoparticles were prepared using CTAB as a structure directing agent, and a mixture of TEOS and bis(triethoxysilyl-propyl)disulfide (BTSPD) as silane source (involving a silane mixture with a molar ratio of 70:30). Promisingly, the nanoparticles broke down at higher glutathione concentrations, where a thiol containing tripeptide reduced the disulfide bonds, causing drug release through the degradation of the particles. These nanoparticles presented a self-destructive behavior and an enhanced drug releasing performance for targeted anticancer drug-release inside glioma C6 cancer cells. These responsive hybrid particles, termed by the authors as “pop goes the particle” (Figure 6), were more cytotoxically effective than non-breakable particles when exposed to a reducing agent. In the same year, Croissant and co-workers prepared biodegradable bridged silsesquioxane nanoparticles, based on disulfide linkers [41]. Different biodegradable disulfide-based nanoparticles were synthesized during the sol-gel process using CTAB as structure directing agent and three different combinations of precursors as silane sources. A bis(trialkoxysilated)disulfide together with a two-photon electron donor or porphyrin-based precursors were used during particle synthesis via co-condensation. Here again, the degradation was proven to occur via the redox cleavage of the disulfide linker with glutathione tripeptides as intracellular bio-reducing agents. These biodegradable disulfide-bridged frameworks were demonstrated to be an efficient biodegradable theranostic tool for cancer treatment. However, in a reported example by Wu et al., the molecule bis[3-(triethoxysilyl]propyl]tetrasulfide was selected as silylated organosilica precursor (containing -S-S-S-S- bonds), to form large-pore ultra-small MONs via the co-condensation reaction with TEOS [42]. The sulfide bonds were used to generate thioether bridged systems uniformly distributed within the organosilica nanoparticles, which could degrade through a redox reaction triggered by glutathione, a tripeptide reducing reagent. Here, cetyltrimethylammonium chloride (CTAC) instead of CTAB was used as surfactant. Monodisperse, ultralarge-radial-pore, flower-like MONs with a diameter of ~30 nm were obtained [17].

The predominant effect of the particle’s porosity and core composition, as well as the particle´s size on the degradation rate was recently demonstrated by the work of Ghandehari et al. [30]. This research group prepared redox-responsive mesoporous disulfide-based nanoparticles with different porosities and sizes (from 58 to 332 nm), containing both disulfide and tetrasulfide bonds. The biodegradation rate was analyzed in the presence of GSH, which was used to mimic the intracellular reducing conditions. Interestingly, the non-porous particles display surface erosion, while the porous ones showed both bulk and surface erosion under these conditions, followed nicely by TEM. The particles with diameters of around 100 nm showed faster degradation. Therefore, that research study showed that a controlled degradation profile promises effective drug delivery towards an established carrier elimination profile.

**Figure 7:**
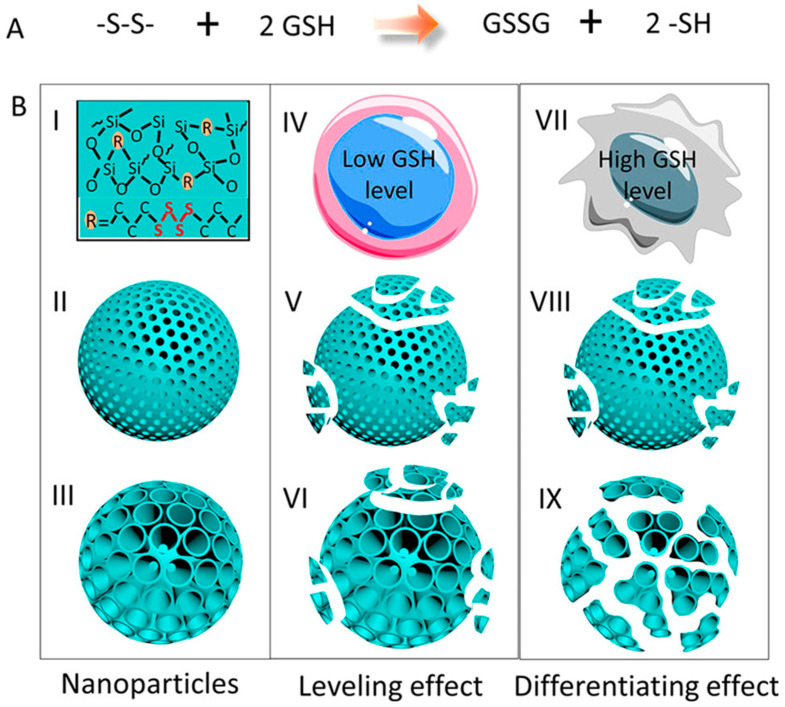
Pore structure-dependent degradability of nanoparticles within normal cells and cancer cells scheme: (**A**) redox reaction of the disulfide bond and GSH; (**B**) illustration of the organic-inorganic hybrid composition of drug delivery MONs, with small or large pores, and its degradation in normal cells with low level of GSH; or within cancer cells containing high level of GSH. Reproduced from [43] (https://doi.org/10.1021/acs.chemmater.6b03896), with permission from the American Chemical Society.

The effect of the particle’s pore size on the degradability was also investigated further by Yang and co-workers with glutathione-responsive biodegradable organosilica nanoparticles for protein delivery [43]. In normal cells, the ones with large pores showed a slower degradation behavior compared to those with small pores, whereas in cancer cells, a much faster degradation rate was shown (Figure 7). Regarding the influence of the particle’s size on their degradation, Yu L. et al. synthesized uniform mesoporous organosilica nanoparticles with sizes less than 50 nm, which had physiologically active thioether bonds incorporated into their framework [44]. These ultra-small hybrid MONs showed a significantly higher reduction-responsive biodegradation rate compared to pure inorganic Si-O-Si containing mesoporous silica nanoparticles. Their biodegradation responsiveness in reductive tumor microenvironments enabled the MONs to induce more specific and improved drug delivery, importantly against cancer.

More recently, a comparative study from Yue et al. compared the features of disulfide containing MONs (here described as ss-MONs) with traditional MSN. Both kinds of particles were synthesized with equal sizes and morphologies utilizing the well-known sol-gel method [45]. In this case, as in many other examples reported, after surfactant removal, the chemotherapeutic drug doxorubicin (DOX) was uploaded into the pores of both systems. Here, notable differences were detected between DOX-loaded ss-MONs and DOX-loaded MSNs in terms of endocytosis, drug release, cytotoxicity, and therapeutic effect. In fact, this ss-MON type was demonstrated to have a higher drug-loading capacity and a higher intracellular drug release at high glutathione concentrations, presented in tumors, causing the breaking of the particle´s framework through the cleavage of disulfide bridges.

**Figure 8:**
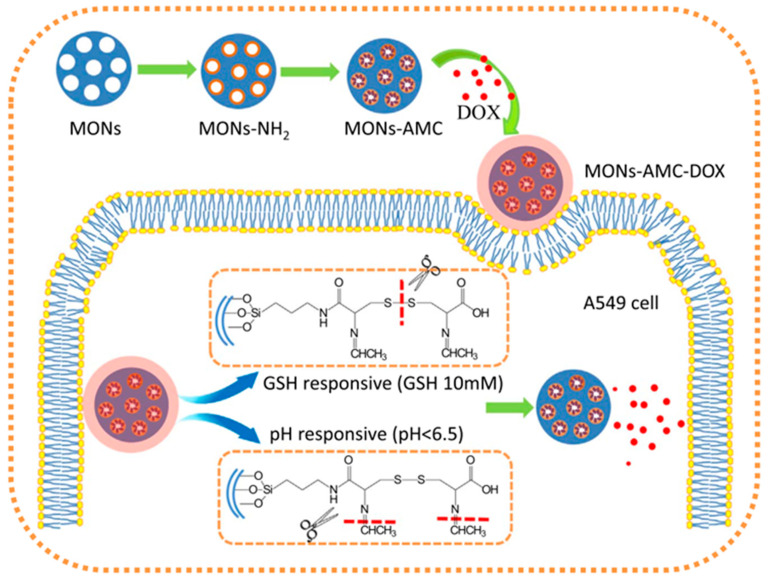
Illustration of the synthesis and release of acetaldehyde-modified-cystine and doxorubicin (DOX)-loaded pH/redox dual responsive hybrid MONs for drug delivery. Reproduced from [46] (https://doi.org/10.1016/j.talanta.2017.07.017), with permission from Elsevier.

In practice, the design and use of multifunctional responsive drug nanocarrier systems, responding to different stimuli, which could enhance the desirable performance of distinguishing better between normal or cancer tissues, is yet challenging. Hu L.-L. et al. prepared dual pH and redox responsive MONs, through the incorporation of both -S-S- bonds and the two -C=N- bonds containing Schiff base acetaldehyde-modified-cystine (AMC) into the nanoparticle’s framework (see Figure 8) [46]. By the creation of a conjugate through electrostatic interactions between DOX and AMC, the model anticancer drug doxorubicin was directly linked to the nanoparticles. Due to the fluorescent nature of AMC, no extra fluorescent labels were needed, providing tracing capabilities to the nanosystem. This kind of material, containing both the -S-S- bond and two susceptible acidic responsive Schiff bonds, was able to degrade in the presence of glutathione within the acidic cell environment.

**Figure 9:**
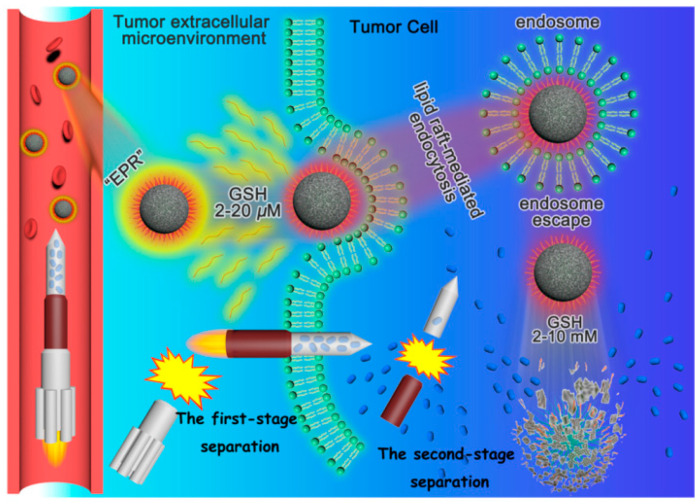
Illustration of the rocket-mimetic organosilica hybrid drug nanocarrier and its two stage redox responsiveness in the tumor microenvironment. Reproduced from [47] (https://doi.org/10.1021/acs.nanolett.9b03340), with permission from the American Chemical Society.

It is well-known that through the grafting of polyethylene glycol (PEG) chains onto the outer silica surface, also known as “PEGylation”, the circulation time of a designed nanosystem in the blood stream is prolonged [38]. Bearing this in mind, Jia X. et al. envisioned a rocket-mimetic strategy for improving tumor accumulation and safety in future cancer chemotherapy [47]. The authors prepared a nanocarrier based on two-stage redox-responsive organosilica nanoparticles, modified using disulfide-bonded PEG and amido-bonded polyethylenimine (PEI) (see Figure 9). If the nanoparticles reach the tumor’s extracellular microenvironment, where low GSH concentrations (2–20 μM) were present, the first response to the reducing environment was triggered, leading to the separation of PEG and to the exposure of PEI. High intracellular GSH concentrations trigger the second stage of the redox-responsive behavior, causing the degradation of the particle’s framework and thus releasing the drug. This innovative nanosystem was prepared using poly(ε-caprolactone)-*b*-poly(acrylic acid) (PCL-*b*-PAA) as a micellar template, and (3-mercaptopropyl)trimethoxysilane acted as a silane source. Carboxyl and thiol groups of the template faced the outside of the micelle. In the next step, the obtained organosilica nanoparticles were functionalized with thiol-modified PEG, through an oxidation of the thiol groups and iodine, leading to disulfide bonds, which are susceptible to high GSH concentrations. This fancy rocket-mimetic drug carrier design displayed a longer circulation in the bloodstream, higher tumor accumulation, and 2.5 times higher efficacy.

### 3.2. MONs Containing Other Interesting Biobreakable Organosilane Bridges

**Figure 10:**
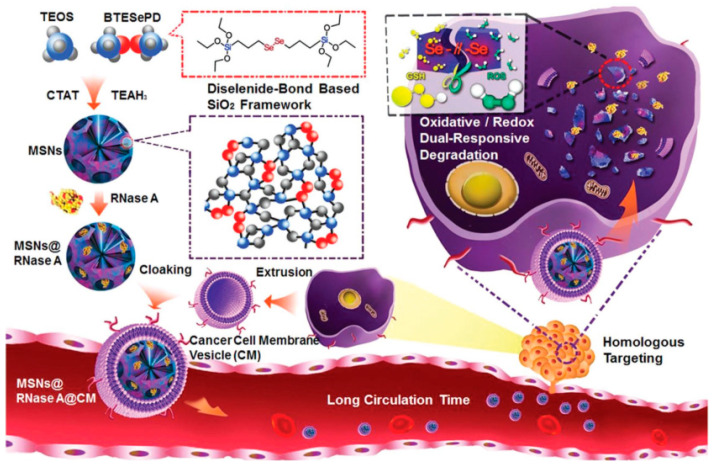
Illustration of the synthesis of biodegradable diselenide-bridged organosilica nanoparticles and its application for dual-responsive, cancer-cell-membrane-mimetic protein delivery. Reproduced from [48] (https://doi.org/10.1002/adma.201801198), with permission from John Wiley and Sons.

The selection of selenide-based alkoxysilane bridges can also be a very promising alternative to commonly selected sulfur-based units, due to an easy cleavable behavior under different oxidative or redox conditions, either by forming seleninic acid or selenol. One of the very few examples of the construction of mesoporous nanoparticles for sensitive drug release using Se-Se bonds was reported by Shao D. and coworkers, who were inspired by the selenium antioxidant properties and proposed an elegant biodegradable organosilica nanosystem prepared to deliver proteins. This dual-responsive material was prepared through the incorporation of diselenide-bond-containing molecules into the nanoparticle’s framework, as seen in Figure 10 [48]. The biodegradation of this particle’s matrix was triggered by redox conditions (i.e., glutathione) and in a media mimicking intracellular ROS (reactive oxygen species) conditions by using H_2_O_2_ in similar concentrations as in a real tumor micro-environment. The particles were synthesized through a sol-gel process using TEOS and the selenide containing bis[3-(triethoxysilyl)propyl]diselenide as silica sources. Particles with a diameter of around 50 nm were obtained and loaded with cytotoxic ribonuclease A (RNase A) within the internal pores through electrostatic interactions. After exposure to oxidative or reducing conditions, the uploaded protein was released through the degradation of the particle’s framework.

**Figure 11:**
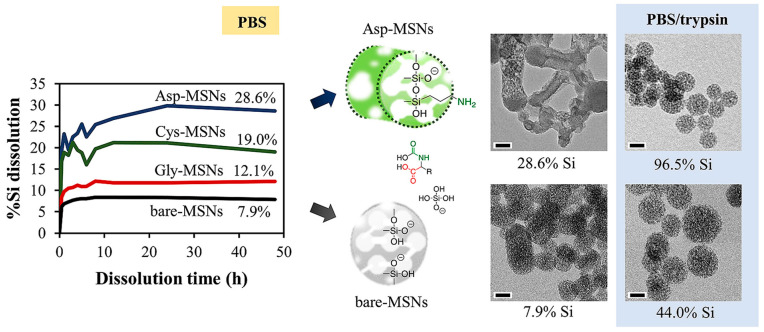
Scheme showing the degradation profile by following the % Si dissolution of bare, Gly- Asp-, and Cys-MONs in PBS at pH 7.4 with the time, and corresponding TEM images in the presence of trypsin enzyme in PBS (pH 7.4) to simulate their degradation in a human system. Reproduced from [49] (https://doi.org/10.1016/j.micromeso.2019.02.033), with permission from Elsevier.

The possibility to tailor the degradation by incorporating functional organic molecules that provide additional hydrolytic sites was recently attempted by Ratirotjanakul and co-workers by using amino acids, the natural building blocks of proteins [49]. For that study, three amino acids, which act as internal biodegradable promotors, were chosen with different side chains, molecular size, polarity, and nucleophilicity: (i) glycine (Gly), (ii) aspartic acid (Asp) and (iii) cysteine (Cys). Their usage within the MONs frameworks was demonstrated to accelerate the degradation through the breaking of the linkage of the amino acid units. In that work, the amino acid moieties were conjugated with (3-isocyanatopropyl)triethoxysilane and reacted with TEOS in a molar ratio of 1:1 to form the MONs. The authors observed that the morphology and the size of these MONs were governed by the hydrophilicity, length, and the amount of the organosilane selected. Here, the presence of amino acids was demonstrated to accelerate the nanoparticle’s biodegradation through the cleavage of the amide and urea bonds formed between the amino acids and the silica framework. MONs containing amino acids with carboxyl side chains (the most nucleophilic side chain) showed the faster particle disintegration (see Figure 11).

**Figure 12:**
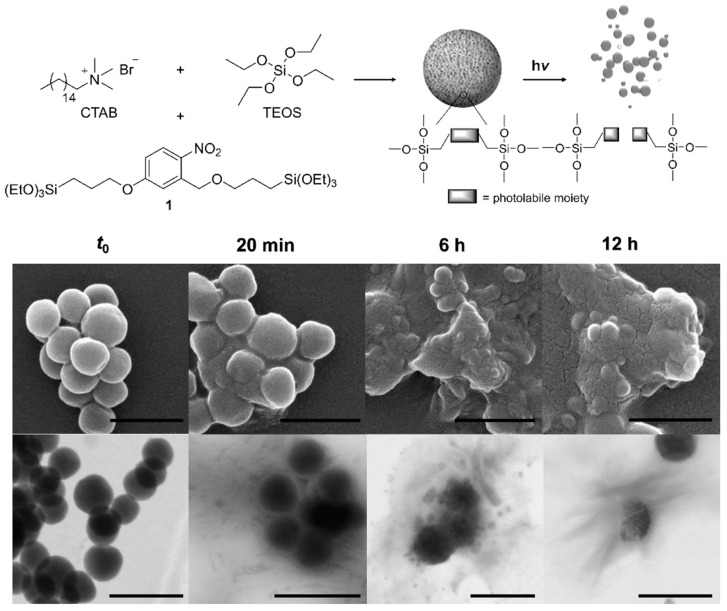
Schematic representation of the preparation and light-induced degradation mechanism, followed by SEM and TEM upon irradiation at λ = 327 nm for 12 h. Adapted with permission from [50] (https://doi.org/10.1021/acs.chemmater.9b03937), with permission from the American Chemical Society.

Among the previous methods mentioned above, degradation triggered upon light exposure is currently a very attractive trigger tool selected for external light-stimulated release applications, which allow an easy and efficient spatiotemporal control. The preparation of light-breakable organo-bridged mesoporous silica nanoparticles was attempted by Picchetti et al., who integrated photocleavable molecules in the silica network through the usage of light-cleavable bis-alkoxysilane linkers [50]. For the particle preparation, a bis-alkoxysilane precursor was used, which contained 2-nitrobenzyl ether groups as photolabile photodegradable moieties (showing an organic content in the particles of around 30 wt.%) able to be cleaved through irradiation with UV light. The cleavage of the groups led to the complete degradation of the silica framework upon exposure to UV light, and then their cargo was released (as seen in Figure 12). In this case, 7-dehydrocholesterol (a natural vitamin D3 precursor) was selected as a hydrophobic test molecule of biological interest, and the release was monitored in the dark and upon irradiation.

### 3.3. Degradable MONs Grafted with Stimuli-Responsive Molecular Gates

**Figure 13:**
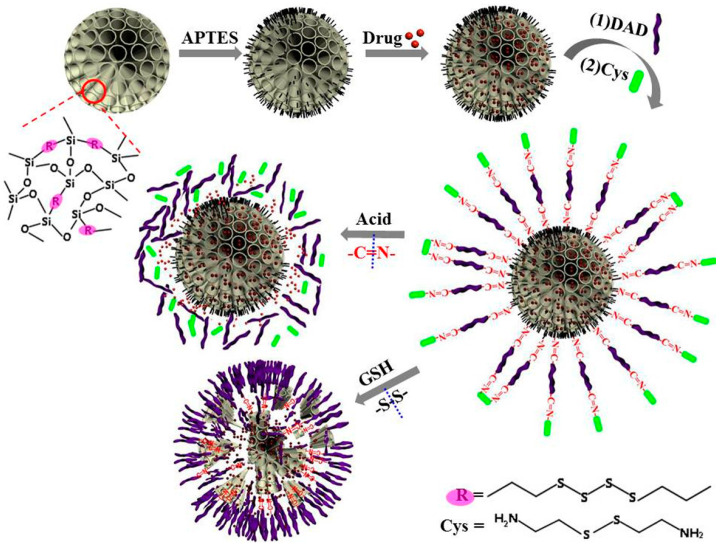
Synthesis, release of doxorubicin and degradation of the sulfide bonds containing organosilica nanoparticles, decorated with a responsive layer that prevented the uploaded drug from leaking out the pores. Reproduced from [53] (https://doi.org/10.1016/j.msec.2020.110914), with permission from Elsevier.

Gated silica mesoporous nanomaterials have mainly been promoted in biomedicine since last two decades for developing new, more efficient and safety improved therapies [51]. For this purpose, certain stimuli-responsive units, which are grafted onto degradable porous silica nanomaterials, acting as gates, are promising candidates for the selective transport of the cargo into the cell or cancer tissues, while avoiding premature degradation or burst release. Besides the temperature, the pH is one of the most studied internal chemical stimuli for drug delivery applications. The fact that pH gradients are present in the cell’s internal parts (pH 6–6.2 in early endosome to pH 5.0–4.5 in lysosome) was explored long ago [52]. Following the idea behind this type of responsive materials, the cargo release from MONs has been exploited via pH-responsive Schiff-base interactions. A very recent example was reported by Li X. et al., who prepared degradable disulfide bonds containing organosilica nanoparticles for dual pH/redox-responsive drug release. Here, cystamine (Cys) was linked with dialdehyde (DAD) dextrin to form a responsive layer that prevented the uploaded drug from leaking out the pores (see Figure 13) [53]. These particles were synthesized by the sol-gel process using TEOS and bis[3-(triethoxysilyl)-propyl]tetrasulfide as silane sources, and the pores were loaded with the model drug doxorubicin hydrochloride. The Schiff base (-N=C-) structure from the responsive layer was able to cleave in the weakly acidic tumor environment, while the S-S bonds incorporated in the particle’s framework lead to GSH-responsive degradation of the organosilica nanoparticles, causing drug release.

**Figure 14:**
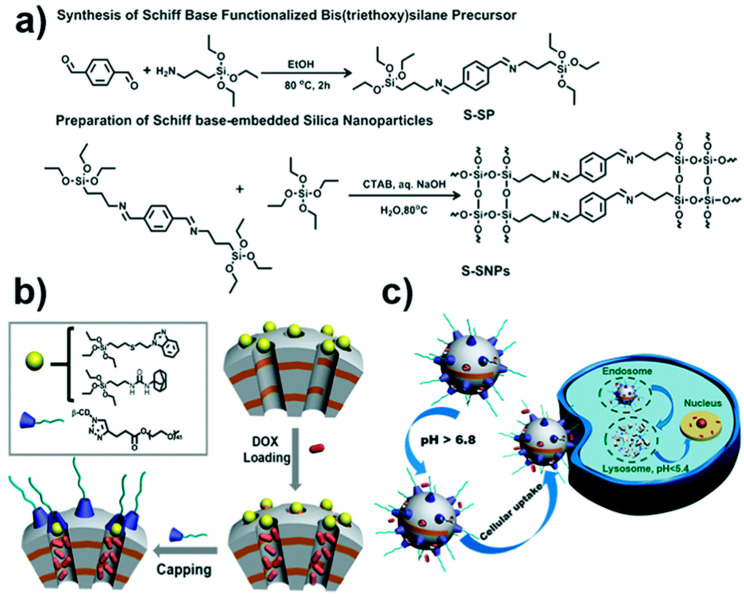
Scheme of the construction of MON-based prodrug nanoparticles synthesized via rationally incorporating benzoic-imine bonds into silicate frameworks (**a**) and functionalized with caps for their applications in controlled drug delivery (**b**). Cellular uptake and efficient controlled drug release due to the acidic pH present within the cancer cell microenvironment, accompanied by the MONs degradability (**c**). Reproduced from [54] (https://doi.org/10.1039/C8CC05043K), with permission from The Royal Society of Chemistry.

Based on the strategy of incorporating Schiff-base components into the nanoparticle’s framework, Liu L. and co-workers also prepared pH-responsive degradable mesoporous organosilica nanocarriers. Here, the authors tested the nanomaterials in vivo as anti-cancer drug delivery vehicles applied for tumors and other key organs [54]. A phenylene Schiff base-bridged organosilane precusor and TEOS were used as silane sources during the base-catalyzed Stöber process, benzoic imine bonds being present in the silica framework (see Figure 14a). As mentioned before in this review, the incorporation of PEG-type hydrophilic polymers is crucial to increase the nanocarrier’s stability, maximizing the “stealth” features and reducing opsonization, all prolonging the blood circulation of the material [55]. Therefore, β-cyclodextrin-ended poly(ethylene)glycol (β-CD-PEG) and benzimidazole (BzI) were utilized for the functionalization of the Schiff-base embedded nanoparticle’s surface to control the release of chemotherapeutic drugs. Through host–guest interactions of pH responsive BzI/β-CD or pH inert adamantane, β-CD-PEG was used as caps for the organosilica nanoparticles. Thus, these nanocarriers were stable under physiological conditions while degrading under acidic conditions, starting from the outer surface (Figure 14b).

## 4. Degradable Periodic Mesoporous Organosilicas (PMOs) for Drug Delivery

**Figure 15:**
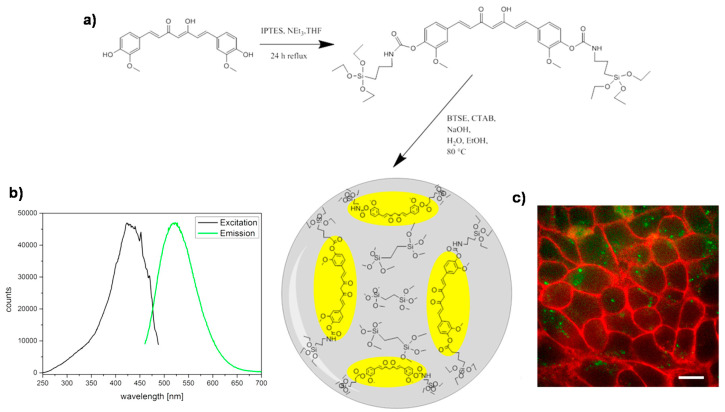
(**a**) Preparation of PMOs (yellow-colored represents the curcumin-based units) without using additional silica; (**b**) Fluorescence excitation and emission spectra of the nanomaterial in PBS buffer; (**c**) Cellular uptake of these PMOs (green) after 24 h on HeLa cells (red: Wheat Germ Agglutinin, WGA 647 membrane staining); scale bar represents 10 μm. Adapted from [56] (https://doi.org/10.1016/j.micromeso.2015.12.006), with permission from Elsevier.

Previous reports have shown the great potential of MONs in drug delivery applications, yet their content of degradable moieties in the framework may hinder their maximum degradation capability and furthermore, their drug encapsulation efficiency. In view of the importance for drug delivery applications, herein, periodic mesoporous organosilicas (PMOs) might be a future strong choice in competition with other highly porous hybrid organic–inorganic nanomaterials, due to their strikingly outstanding high number of organic moieties building the silica framework. Despite the vast majority of PMOs described in the literature, which often used TEOS for their preparation, few examples are genuinely synthesized with only organosilica units acting as bridges building the silica framework, hence here highlighted. Without doubt, their extremely interesting features are also confronted with their difficult preparation, which is not as straight forward as the classical MSNs or even the MONs. A number of fundamental parameters have to be taken into consideration concerning the attempted final morphology, degradability, and thus, their desired applications. Like for MONs, the precursor’s length and flexibility, the degradable nature of the anchoring groups within the organosilica-bridges, the number of the functional groups and their homogeneous distribution within the PMOs walls, all while maintaining an overall porous periodicity, have to be taken into major account. The successful preparation of fully organosilane-containing PMOs will be of high importance for future drug delivery purposes in nanomedicine. For instance, the PMOs prepared by S. Datz and co-workers consisted exclusively of ethane organic bridging groups [56]. A curcumin-based precursor was introduced during the mesoporous organosilica nanoparticle’s synthesis, attached through carbamate bonds. Here, 3-isocyanatopropyl-(triethoxy)silane (see Figure 15a) was used as a silane source, the fluorescent nature of added tracking features to the nanoparticles. Interestingly, the incorporation of this molecule as the only organic constituent did not negatively affect the framework stability. The presented high porosity obtained led to a promising drug delivery system. The pores of these fluorescent organosilica nanoparticles (Figure 15b) were coated with a lipid bilayer to avoid uncontrolled leaking of loaded rhodamine B, and the release was tested in in vitro studies. Potentially, the whole nanosystem was proven to be internalized inside HeLa cells (as seen in Figure 15c).

Compared to conventional mesoporous silica nanomaterials, the loading and release of confined guest or therapeutic molecules from the pores of PMOs can be controlled through the hydrophobic interactions created with the huge content of organic moieties placed inside their pores walls [57]. This special feature owned by PMOs might avoid the additional use of molecular gates or capping agents grafted covalently on the silica surface to seal such pores. Therefore, dual responsiveness can be suggested as a promising feature for the design of degradable PMOs for selective drug delivery applications. Based on that concept, Lu J. et al. synthesized pH/glutathione dual responsive disulfide-bridged mesoporous organosilica nanoparticles. Folic acid (FA) decorated bovine serum albumin (BSA) was used to improve biocompatibility and add targetability [58]. The nanoparticles were prepared by using (3-mercaptopropyl)trimethoxysilane (MPTMS) and 3-thiocyanatopropyltriethoxysilane (TPTES) as the only silane sources used for the sol-gel process. Through a nucleophilic substitution reaction, internal disulfide bonds were linked to the silica framework. Because of the mesoporous structure, GSH was able to go into the particle’s framework, causing the breakdown of the particles. In this case, the surface of the organosilica nanoparticles was modified using folic acid-decorated bovine serum albumin, enhancing the biocompatibility of the nanoparticles. In normal physiological conditions, BSA covered the surface, preventing premature drug leakage. However, at acidic pH, the electrostatic interactions were disrupted, hence BSA was detached and the drug released. Through the folic acid molecules, the drug can be selectively delivery into the tumor tissue.

Besides the use of commonly selected degradable sulfide-based units, Omar et al. also investigated biodegradable mesoporous organosilica nanoparticles for controlled drug release. This research group prepared silica nanoparticles with cleavable azobenzene linkers distributed in the particle’s framework [59]. Through a reaction of azo-based linkers with benzene or ethane, bridging groups were created, which are useful during the sol-gel process. CTAB was used as a surfactant and a condensation reaction of the azo-based precursor and 1,4-bis-(triethoxysilyl)benzene or 1,2-bis-(trimethoxysilyl)ethane was conducted. The on-demand drug delivery took place through a reductive cleavage of the azo-bond through azoreductase, making this system interesting for colon-specific drug delivery systems [60].

**Figure 16:**
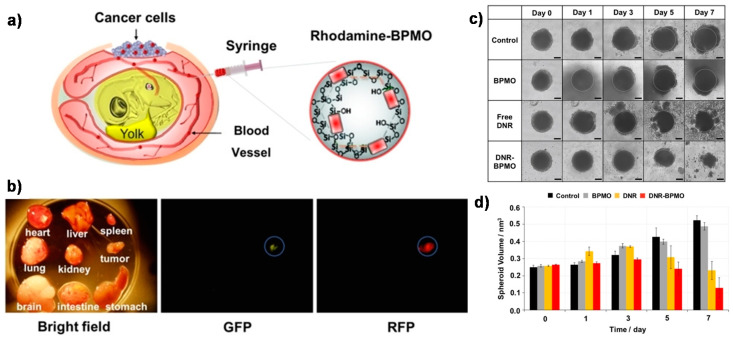
Schematic illustration of (**a**) chicken egg tumor model injected by rhodamine B-labeled BPMO nanoparticles (0.2 mg. 100 μL^−1^) intravenously into chicken egg blood vessel. (**b**) Bright field and fluorescence images indicate the preferential accumulation of the BPMOs in the tumor, expressing green or red fluorescent protein (GFP and RFP, respectively); (**c**) Optical image of ovarian tumor spheroid; (**d**) calculated spheroid volume after treatment with no injection (Control), free BPMO nanoparticles, free daunorubicin (DNR) or DNR-BPMO nanoparticles over a period of 7 days. Scale bar: 100 μm Reproduced from [63] (https://doi.org/10.1002/cmdc.201900595) with permission from Wiley-VCH.

Through the careful selection of biodegradable bridges inserted between the two Si atoms and locating them within the channel walls, the properties of the PMOs can be tailored towards envisioning future clinical applications. The in vitro studies of their potential degradability demonstrated that the complete elimination from animal models might be expected. Furthermore, PMOs can be designed to present maximum cargo uptake efficiency without the extra usage of stimuli-responsive gates to block the pores, by possible interactions between the drug units and the organic components created in the nanoparticle’s surface [61,62]. Very recently, other promising biodegradable periodic mesoporous organosilica (BPMO) nanoparticles were prepared by using tetrasulfide units [63]. These particles potentially degraded under reducing conditions at higher glutathione concentrations. Remarkably, the authors opted for modifying the silica surface with phosphonate derivatives, in order to enhance the nanoparticle’s dispersion in solution. Here, the pores of the nanoparticles were uploaded with daunorubicin (DNR), a chemotherapeutic drug, chosen to prove their applicability for cancer treatment, and the material showed potential biological activity on both tumor spheroids and chicken egg tumor models (Figure 16).

## 5. Conclusions

Among different synthetic platforms, silica-based nanomaterials have set a precedent in terms of controlled drug release. The evolution of new administration mechanisms has to deal with biodistribution, pharmacokinetics and cell uptake control. Nevertheless, this is a major hurdle in nanomedicine reaching the clinic, and it has become commonly accepted in the community that the development of fully biodegradable materials is required to avoid the concerns of accumulation of retained materials, in particular for long-term use or high-dose therapies such as chemotherapy. Unlike conventional mesoporous silica nanoparticles, mesoporous organosilica nanostructures introduce organic functional groups in the walls as bridges, providing more efficient degradable nanomedicine solutions. As described in this mini review, studies reported in the literature demonstrated the importance of the right choice of organic cleavable bridges and their homogenous distribution in the silica structure, which can endow degradability to some extent. While redox or enzymatic cleavage has been the most selected approach for the construction of biodegradable organosilica nanoparticles by inserting responsive disulfide or oxamide-based alkoxysilane bridges inside the silica framework, there have been other emerging potential degradable units. For example, diselenide, aminoacids, azobenzene, phosphazenes or even photocleavable molecules have blossomed in recent years as even more attractive linkage alternatives towards facile degradation. Furthermore, the concept of on-demand gated-nanomaterials has been promoted for developing more efficient and safe improved therapies while avoiding premature degradation or burst cargo release. Therefore, the well-known efficiency of sol-gel chemistry is paving the way to new possible designs of organosilica nanomaterials, yet aiming to preserve a degradable behaviour to allow the prospering enhancement of the conventional silica-based mesoporous nanosystems towards their final clinical purposes. The synthesis of mesoporous silica materials without any additional silica source (e.g., TEOS) only using organosilica precursors may be an important challenge for scientists in the future. This will make these materials even more promising for biomedical applications, due to the tenability of the degradation of the nanoparticles through the utilization of suitable organosilane precursors.
